# Inhibition of Colon Cancer Recurrence via Exogenous TRAIL Delivery Using Gel-like Coacervate Microdroplets

**DOI:** 10.3390/gels8070427

**Published:** 2022-07-08

**Authors:** Sungjun Kim, Yerim Jwa, Jiyeon Hong, Kyobum Kim

**Affiliations:** Department of Chemical & Biochemical Engineering, Dongguk University, 30, Pildong-ro 1-gil, Jung-gu, Seoul 22012, Korea; sungjun.kim@dgu.ac.kr (S.K.); jwayerim@naver.com (Y.J.); 3stellar@naver.com (J.H.)

**Keywords:** TRAIL, coacervate, colon cancer, cancer recurrence, drug delivery system

## Abstract

Colon cancer (CC) belongs to the three major malignancies with a high recurrence rate. Therefore, a novel drug delivery system that can prevent CC recurrence while minimizing side effects is needed. Tumor-necrosis-factor-related apoptosis-inducing ligand (TRAIL) has recently been spotlighted as a protein drug that can induce apoptosis of cancer cells specifically. However, its short in vivo half-life is still a challenge to overcome. Hence, in this study, a gel-like mPEGylated coacervate (mPEG-Coa) delivery platform was developed through electrostatic interaction of mPEG-poly(ethylene arginylaspartate diglyceride) (mPEG-PEAD) and heparin for effective protection of cargo TRAIL, subsequently preserving its bioactivity. mPEG-Coa could protect cargo TRAIL against protease. Sustained release was observed for a long-term (14 days). In addition, recurrence of HCT-116 cells was suppressed when cells were treated with TRAIL-loaded mPEG-Coa for 7 days through long-term continuous supply of active TRAIL, whereas re-proliferation occurred in the bolus TRAIL-treated group. Taken together, these results suggest that our gel-like mPEG-Coa could be utilized as a functional delivery platform to suppress CC recurrence by exogenously supplying TRAIL for a long time with a single administration.

## 1. Introduction

Colon cancer (CC) is caused by transformation of normal colonic epithelium into adenomatous polyps. It is closely related to various factors such as lifestyle, diet, and genetic mutations [1]. CC is one of the three major malignancies along with breast cancer and lung cancer. It is ranked third in incidence and second in mortality [2]. Additionally, according to GLOBOCAN 2020 estimates, there will be more than 1.9 million new cases of CC worldwide and 935,000 deaths in 2020 [3]. Currently available treatments for CC include surgery, radiation therapy, and chemotherapy [4]. However, surgery could remove healthy colon tissues during tumor resection or cause pain. Radiation therapy could induce infertility problems in patients. Moreover, non-specific delivery of chemodrugs to off-target sites can ultimately cause unwanted side effects such as gastrointestinal toxicity, liver toxicity, and hematologic disorders [5,6]. Notably, tumor recurrence occurs in 30–50% of CC patients after chemotherapy [7]. In addition, considering patient’s condition, systemic chemotherapy and radiation therapy are performed at least 2–3 weeks after surgery However, the golden hour to remove residual cancer cells is often missed during this period [8]. Therefore, the importance of developing effective and safe treatment techniques to overcome problems of tumor recurrence in CC has been emphasized.

Tumor-necrosis-factor-related apoptosis-inducing ligand (TRAIL) could be an alternative protein drug to treat CC. It binds to cell surface receptors TRAIL-R1 and TRAIL-R2 and triggers an extrinsic apoptotic pathway [9,10]. Condidering selective apoptosis-inducing properties of TRAIL for cancer cells and high expression profile of TRAIL receptors in cancerous cells, TRAIL is considered a very attractive clinical therapeutic candidate [10,11]. However, active clinical translation attempts by a simple injection of bolus TRAIL have been limited due to its very short in vivo half-life and inefficient delivery with insufficient therapeutic outcomes [12]. Maintaining high concentrations of TRAIL at the tumor site usually requires repeated dosing, which may increase drug resistance and induce potential toxicity. Therefore, the development of a novel delivery system that enables higher bioavailability and sustainability of TRAIL to continuously suppress CC with a single administration is required.

Coacervate (Coa) is a self-assembled gel-like colloidal droplet formed by the association between polycation and polyanion by Coulombic force [13,14]. In particular, poly(ethylene arginylaspartate diglyceride) (PEAD) (i.e., polycation) can interact with heparin (i.e., polyanionic counterpart) by electrostatic interaction in aqueous conditions, forming a gel-like Coa structure. This Coa can be used as an exogenous growth factor (GF) delivery carrier for various tissue engineering applications including osteochondral tissue regeneration [15], bone regeneration [13,16], vascular regeneration [17], and repair of damaged skin tissues [18,19]. However, random aggregation of Coa has put the brakes on intravenous administration for clinical use. Thus, methoxy-poly(ethylene glycol) (mPEG) was conjugated on the PEAD cation backbone to augment colloidal stability of Coa in our previous studies [20,21]. The PEG moiety of mPEGylated PEAD (mPEG-PEAD) could provide a shielding effect to Coa droplets by blocking access to unwanted surrounding molecules. Therefore, mPEG-Coa formed with mPEG-PEAD and heparin had superior colloidal stability than Coa without PEG modification, consequently exhibiting excellent functionality as a GF delivery carrier [20,21].

In this study, we evaluated functionalities of mPEG-Coa as an exogenous TRAIL delivery carrier for cargo TRAIL protection and long-term sustained supply to inhibit CC cell recurrence. TRAIL was incorporated into gel-like mPEG-Coa droplets and HCT-116 cells (i.e., CC cells) were cultured for a long period (7 days) with TRAIL-loaded mPEG-Coa. Specifically, the present study had the following objectives: (1) to investigate in vitro cancer-killing efficacy of TRAIL on HCT-116 cells, (2) to determine the cargo protection ability of mPEG-Coa and sustained cargo release kinetics, and (3) to determine the ability of mPEG-Coa-mediated TRAIL delivery to inhibit tumor recurrence.

## 2. Results and Discussion

### 2.1. In Vitro Anticancer Efficacy of TRAIL

**Figure 1:**
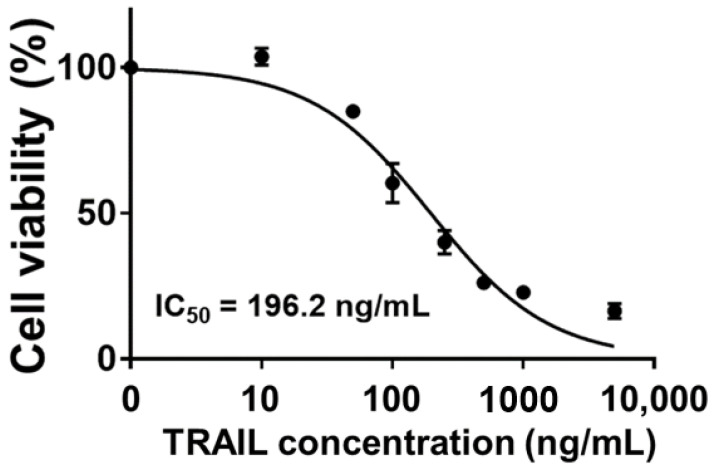
TRAIL-concentration-dependent cell viability of HCT-116 and determined IC_50_ value.

TRAIL is a well-known TNF family member that can induce apoptosis of cancer cells without showing toxicity to normal cells [22]. Binding of TRAIL to death receptor (DR) 4 or DR5 can activate caspase-8, which in turn stimulates caspase-3. Thus, a caspase-mediated extrinsic apoptosis pathway is triggered, leading to cancer cell death. Furthermore, TRAIL triggers another intrinsic apoptosis mechanism. Caspase-8 activated by TRAIL induces cytochrome C release from mitochondria. Released cytochrome C then promotes the formation of apoptosome, leading to caspase-9-mediated apoptosis [23,24]. Due to these characteristics, TRAIL is considered as an alternative anticancer drug candidate. In this study, HCT-116 cells exhibited TRAIL-concentration-dependent cell death. Specifically, at a concentration of 10 ng/mL or less, TRAIL had no significant effect on cell viability. Its IC_50_ value was 196.2 ng/mL (Figure 1). Additionally, a previous study has demonstrated that HCT-116 cells show higher expression levels of both DR4 and DR5 than A549, HT-29, and Jurkat cells. Higher TRAIL-sensitive apoptosis was correspondingly observed [25]. This higher sensitivity of HCT-116 cells to TRAIL compared to HT-29, SW480, and SW620 cells has also been observed in other studies [26]. Consequently, in this study, HCT-116 was selected as a target for TRAIL-mediated anticancer treatment and used in subsequent experiments.

### 2.2. Characterization of mPEG-PEAD and mPEG-Coacervate

**Figure 2:**
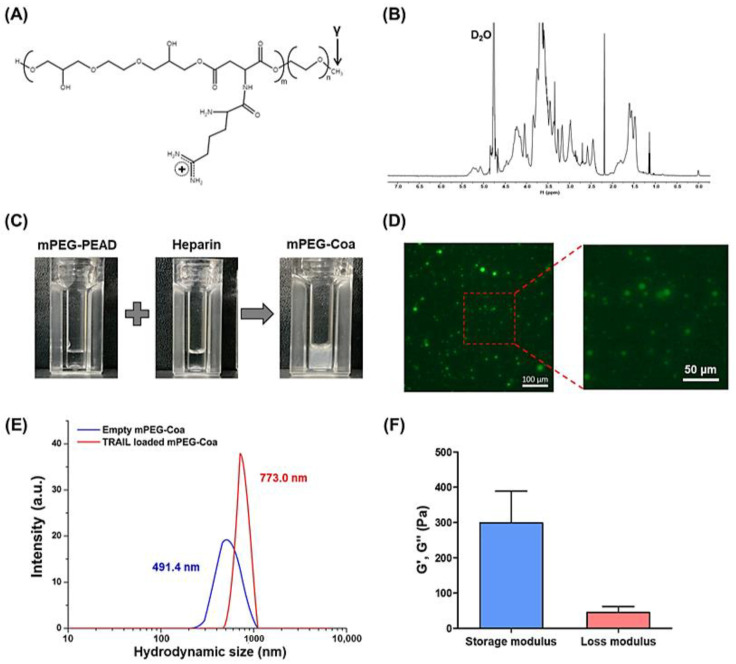
Characterization of mPEG-PEAD and mPEG-Coa. (**A**) Chemical structure of mPEG-PEAD and (**B**) structural analysis using ^1^H-NMR. (**C**) Macroscopic observation of mPEG-PEAD, heparin, and mPEG-Coa and (**D**) fluorescence image of mPEG-Coa. (**E**) Hydrodynamic size of empty mPEG-Coa and TRAIL-loaded mPEG-Coa. (**F**) Viscoelastic properties of mPEG-PEAD.

mPEG-PEAD was synthesized by grafting arginine onto mPEG- Fmoc-poly(ethylene aspartate diglyceride) (mPEG-PED) as previously reported [20,21]. The primary amine of mPEG-PED can be conjugated with various carboxylic acid derivatives, among which arginine is a cationic and biocompatible amino acid (Figure 2A). Structure of synthesized mPEG-PEAD was confirmed by ^1^H-NMR (Figure 2B): 3.4–3.9 ppm (protons of glyceride moiety), 4.3 ppm (protons of aspartate), 3.2 ppm (protons of mPEG), and 1.4–1.7 ppm (protons of arginine). Although the development of drugs and gene delivery systems using cationic polymers has been widely investigated, their toxicity is still a problem to be overcome [14]. Polycations could disrupt cell membranes through interactions with negatively charged cell membranes, causing high cellular toxicity. Meanwhile, high in vitro and in vivo biocompatibility of our PEAD-based Coa has been demonstrated in a series of previous studies [15,16,20,21]. Next, mPEG-PEAD and heparin were prepared by being dissolved them in PBS at a concentration of 1 mg/mL and then mixed at a 1:8 mass ratio. Both mPEG-PEAD and heparin solutions were transparent before mixing. They immediately changed to turbid solutions after mixing, indicating that coacervation (i.e., liquid–liquid phase separation) was successfully induced by the electrostatic interaction between mPEG-PEAD and heparin (Figure 2C). Moreover, mPEG-Coa revealed a stable spherical shape (Figure 2D). Particle size of empty mPEG-Coa (i.e., without cargo TRAIL) was 491.4 nm. The size of TRAIL-loaded mPEG-Coa was 773.0 nm (Figure 2E). The size of mPEG-Coa was slightly increased by TRAIL encapsulation. Viscoelastic properties of mPEG-Coa were measured by rheometer and mPEG-Coa exhibited a higher storage modulus than loss modulus (Figure 2F). Specifically, the storage modulus was 298.8 Pa and the loss modulus was 45.4 Pa, demonstrating gel-like properties of mPEG-Coa. In addition, mPEG-Coa exhibited a zeta potential of −0.2 mV before TRAIL loading and −0.1 mV after TRAIL loading. The near-neutral charge of these mPEG-Coa complexes could prevent electrostatic-interaction-mediated nonspecific adsorption with surrounding serum proteins, thereby avoiding unwanted interference, especially in a physiological environment. Moreover, particles with negative or neutral surface charge could avoid cellular uptake and achieve long blood circulation [27]. In addition, mPEGylation on PEAD provides excellent colloidal stability of the Coa structures. In our previous study, hydrophilic mPEG moiety was incorporated into cationic PEAD backbone. Subsequently obtained mPEG-Coa exhibited improved structural stability, avoiding random aggregation compared to Coa without mPEG modification, thereby increasing GF delivery efficiency and promoting cell activation and downstream differentiation [20,21]. Therefore, our mPEG-Coa could be utilized as an effective anticancer drug delivery system.

### 2.3. Cargo TRAIL Release Phenomenon

**Figure 3:**
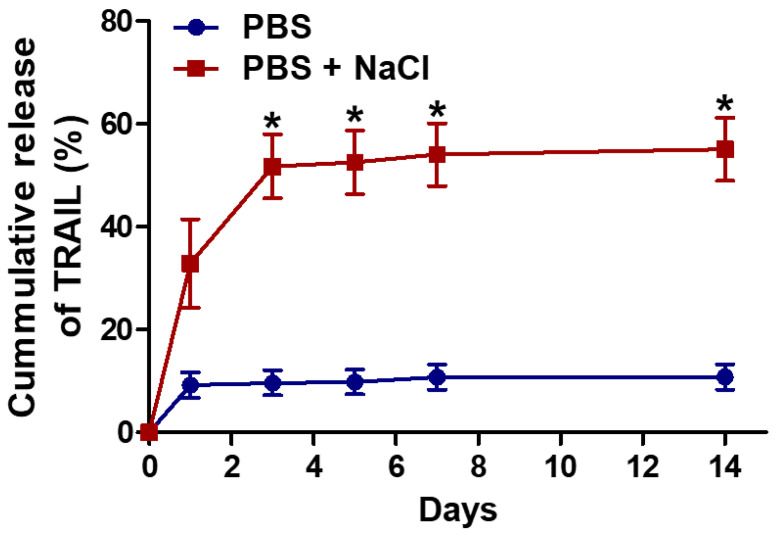
Release profile of TRAIL from mPEG-Coa in PBS (135 mM NaCl) and PBS supplemented with additional NaCl (total 185 mM NaCl). (*) indicates a significant difference as compared with PBS group.

The initial loading efficiency of cargo TRAIL in mPEG-Coa was 88.1%. Its release profile was determined by ELISA. In particular, NaCl-concentration-dependent modulation in the release kinetics of cargo TRAIL was observed (Figure 3). In PBS condition, a sustained TRAIL release pattern was observed and 10.9% of cumulative release was recorded for 14 days. However, in the presence of additional salts (i.e., PBS with 50 mM NaCl), a significantly burst release was observed. Specifically, 36.8% of TRAIL was released in this condition on day 1, whereas 59.4% of TRAIL release was achieved at 14 days. Release kinetics of cargo proteins from Coa predominantly depended on hydrolysis of polycation backbones in the aqueous environment and the binding affinity level (i.e., dissociation constant) between cargo and heparin [14]. Moreover, the structural stability of Coa is closely related with dissociation of cargo protein and further release into the surroundings. Adding electrolytes Na^+^ and Cl^−^ into an aqueous environment where coacervation occurs and subsequent changes in surrounding ionic strength could interfere with the stable electrostatic interaction between mPEG-PEAD, heparin, and TRAIL, which could disrupt the formation of colloidal Coa structure, sequentially accelerating the release of cargo TRAIL from mPEG-Coa. Our previous studies [20,21] have shown dissociation and deformation of Coa structure by manipulating ionic strength. In these studies, Coa without mPEGylation aggregated within 24 h, whereas significant colloidal stability (i.e., less aggregation and retention of spherical structure) was observed in mPEG-Coa. A relatively higher sodium concentration in the tumor microenvironment (TME) [28] could also imply the following sequential processes in physiological conditions upon administration: (1) rapid structural disruption of mPEG-Coa, (2) sufficient release of cargo TRAIL from mPEG-Coa, and (3) preservation of optimal therapeutic efficacy of released TRAIL to suppress cancer cells.

### 2.4. Cargo Protection Ability of mPEG-Coa

**Figure 4:**
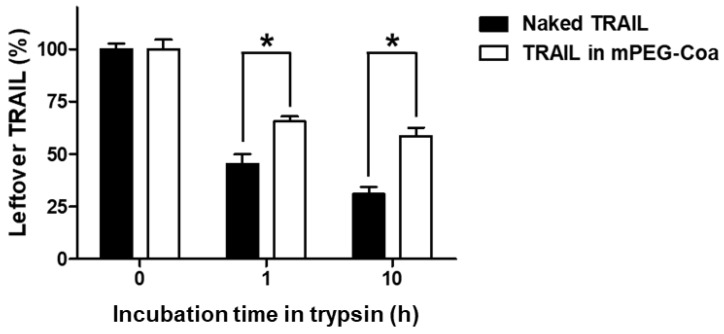
Cargo TRAIL protection ability of mPEG-Coa against trypsin, indicated by the remaining TRAIL amounts. (*) indicates that the two experimental groups are statistically different to each other.

An ideal drug delivery system should also be able to protect the cargo molecule from the stimuli in external environments. In particular, TRAIL has a very short in vivo half-life (3–5 min in mice and 30–60 min in primates) [12,29]. Consequently, in most studies, TRAIL administered directly into the blood vessel is degraded before functioning. High-dose and/or multiple administrations of TRAIL are required to achieve the intended therapeutic efficacy. Therefore, the development of a delivery carrier with sufficient protective capability for incorporated cargo drugs until it reaches the target tumor is necessary. To evaluate the cargo protection ability of mPEG-Coa, naked TRAIL and TRAIL-loaded mPEG-Coa were incubated with trypsin for 10 h (Figure 4). As a result, after 10 h of incubation with trypsin, 31.0% of naked TRAIL without any protection remained. On the other hand, in the TRAIL-loaded mPEG-Coa group, 58.6% of cargo TRIAL was preserved by the protection of mPEG-Coa carriers. These results demonstrat that our mPEG-Coa could efficiently protect cargo TRAIL from the harsh external environment containing protease. Previous studies have also demonstrated similar results for Coa-mediated protection of cargo fibroblast growth factor-2 or interleukin-12 and preservation of their bioactivities in the presence of trypsin [30,31]. Collectively, with a differentiated release profile (Figure 3), it could be anticipated that the protective efficacy of mPEG-Coa (Figure 4) could improve therapeutic outcomes of exogenous TRAIL delivery in TME with high levels of extracellular proteolytic enzymes [32].

### 2.5. Inhibition of Tumor Recurrence via mPEG-Coa-Mediated TRAIL Delivery

**Figure 5:**
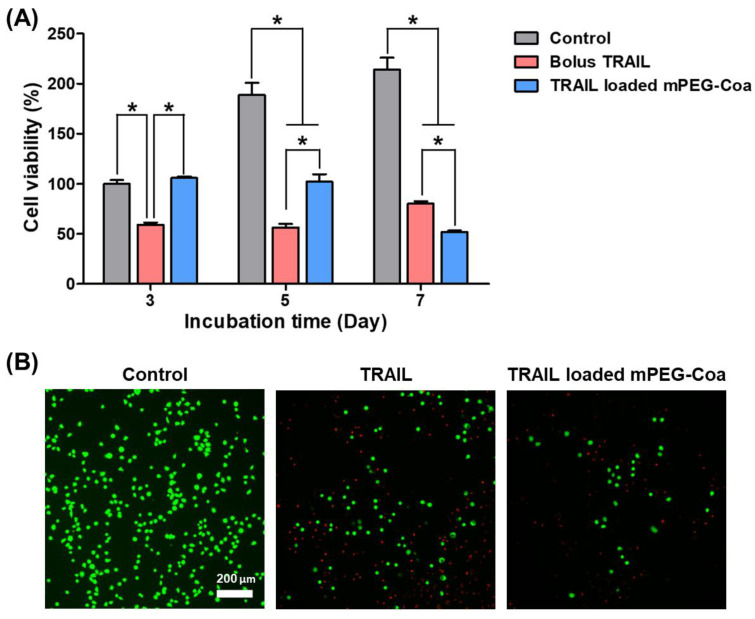
Inhibition of colon cancer recurrence via mPEG-Coa-mediated TRAIL delivery. (**A**) Viability of HCT-116 cells and (**B**) live/dead fluorescence microscope images after 7 days of treatment. (*) indicates that the experimental group is statistically different to indicated experimental groups.

To verify the inhibition of CC cell recurrence by mPEG-Coa-mediated TRAIL delivery, HCT-116 cells were cultured for 7 days with different TRAIL treatment conditions (Figure 5). In the bolus-TRAIL-treated group, significant cancer cell death was observed on days 3 and 5 (Figure 5A). However, cancer recurred and the survival rate of HCT-116 cells was higher at 7 days than that at 5 days. Since a small amount of TRAIL was released from mPEG-Coa in the early stage (i.e., 9.7% cumulative release for 3 days in PBS, Figure 3), suppression of cancer cell proliferation did not appear in the TRAIL-loaded mPEG-Coa group at day 3. The amount of released TRAIL might not reach a sufficient therapeutic dose. On the other hand, the proliferation of HCT-116 cells was no longer observed from day 5. In addition, the viability of HCT-116 cells at day 7 was significantly lower in the mPEG-Coa group than that in the bolus TRAIL treatment group. These results are closely related to the therapeutic threshold and available dose of TRAIL in the surrounding microenvironment. In the bolus TRAIL group, the initially available TRAIL dose was relatively high, showing high anticancer efficacy (i.e., downregulated proliferation of CC cells) until day 5. However, due to the short half-life of bolus TRAIL, it was speculated that cancer could recur at a later stage because the concentration of bioactive TRAIL remained below the therapeutic threshold. Moreover, live/dead fluorescence images exhibited high cytotoxicity in the TRAIL-loaded mPEG-Coa treatment group (Figure 5B). Fewer viable cells (i.e., green cells) were observed in HCT-116 cells treated with TRAIL-loaded mPEG-Coa than in the TRAIL-treated group. In addition, since the completely dead cells were removed during the washing process, even red stained cells were observed to decrease in the TRAIL-loaded mPEG-Coa group. A similar phenomenon was revealed in our previous study [33]. As previously described, mPEG-Coa is capable of inducing a sustained release (Figure 3) while protecting the cargo TRAIL against the external environment (Figure 4). Therefore, a continuous supply of therapeutically available TRAIL could effectively suppress cancer recurrence, although the initial release amount of TRAIL was low in mPEG-Coa mediated TRAIL treatment and significant anticancer effects were not observed until day 3. Taken together, the mPEG-Coa-mediated TRAIL delivery system could (1) protect cargo TRAIL in the tumor microenvironment (TME) with high levels of extracellular protease, (2) continuously supply TRAIL in TME with high levels of NaCl, and (3) subsequently induce cancer cell death and prevent recurrence of cancer cells. Therefore, direct local administration of TRAIL-loaded mPEG-Coa could effectively induce cancer cell death and inhibit recurrence of remaining CC after cancer resection surgery with less systemic toxicity in normal tissues.

## 3. Conclusions

In this study, we investigated inhibition of CC recurrence through mPEG-Coa-mediated exogenous TRAIL delivery. mPEG-Coa (a gel-like colloidal droplet) was self-assembled through electrostatic interaction with mPEG-PEAD and heparin in aqueous environments. During this fabrication process, TRAIL was effectively encapsulated into mPEG-Coa. mPEG-Coa exhibited (1) high TRAIL-loading efficiency of over 88%, (2) protective efficacy of cargo TRAIL in regards to the harsh external environment, and (3) sustained release profile of entrapped TRAIL. Interestingly, in the presence of additional electrolytes such as NaCl, an abrupt release pattern of TRAIL was observed due to changes in ionic strength in the surrounding environment and subsequent interference in the mPEG-PEAD:heparin complex. The treatment of bolus TRAIL in HCT-116 cells resulted in early cancer death due to the highly available TRAIL amount, whereas cancer reproliferated after 7 days since the bioactivity of bolus TRAIL decreased over time. When TRAIL-loaded mPEG-Coa was administered to HCT-116 cells, cancer recurrence was inhibited from 5 days. On day 7, it showed a more significant cancer inhibitory effect than bolus TRAIL. In conclusion, our gel-like mPEG-Coa-based TRAIL delivery system could be utilized as a novel protein drug delivery platform to suppress the recurrence of residual cancer after cancer resection or chemotherapy by continuously supplying available TRAIL.

## 4. Materials and Methods

### 4.1. Materials

6× His-TRAIL was obtained from UBPBio. Mouse Anti-Human TRAIL-UNLB and mouse Anti-Human TRAIL-BIOT (Dallas, TX, USA) were purchased from SouthernBiotech (Birmingham, AL, USA) for ELISA. Dulbecco’s Modified Eagle Medium (DMEM), penicillin–streptomycin, fetal bovine serum (FBS), trypsin, and phosphate-buffered saline (PBS) were purchased from Corning (Corning, NY, USA). Heparin and Bacteroides Heparinase II were received from Selleckchem (Radnor, PA, USA) and New England Biolabs (Beverly, MA, USA), respectively. EZ-Cytox was purchased from DoGenBio (Seoul, Korea). Fmoc-Asp and Fmoc-Arg were obtained from BOC Science (Shirley, NY, US). Monomethoxy polyethylene glycol 750, 1-ethyl-3-(3-dimethylaminopropyl) carbodiimide (EDC), N-hydroxy succinimide (NHS), tetrabutylammonium bromide (TBAB), 4-dimethyl amino pyridine (DMAP), dimethyl formamide (DMF) (anhydrous), 1,4-dioxane, piperidine, 3,3′,5,5′-Tetramethylbenzidine (TMB), and streptavidin-HRP conjugate were obtained from Sigma-Aldrich (St. Louis, MO, USA). Ethylene glycol diglycidyl ether (EGDE) was obtained from TCI (Tokyo, Japan). Protease inhibitor and Live and dead staining kit were obtained from ThermoScientific (Waltham, MA, USA).

### 4.2. In Vitro Anticancer Efficacy of TRAIL

HCT-116 colon cancer cells were cultured in a growth media consisting of 89% (*v/v*) DMEM, 10% (*v/v*) FBS, and 1% (*v/v*) penicillin-streptomycin and incubated at 37 °C with 5% CO_2_ and 95% humidity. When cell confluency reached about 80%, cells were transferred to 96-well cell culture plates at a density of 10,000 cells/well and incubated for 24 h. After 24 h, old media were replaced with fresh media containing 0–5000 ng/mL of TRAIL and incubated for 24 h. After 24 h of TRAIL treatment, old media were discarded and WST-1 reagent was prepared by mixing EZ-Cytox and growth media at a 1:10 (*v/v*) ratio was added to each well. Treated cells were incubated at 37 °C for 3 h. Optical density was then measured at 440 nm by microplate spectrophotometry. Cell viability was calculated with the following equation: Cell viability (%) = (OD value of experimental group—OD value of blank group)/(Average OD value of control group—OD value of blank group) × 100%.

### 4.3. Synthesis of mPEG-PEAD

Poly(ethylene Fmoc-aspartate diglyceride) (FPED) intermediate polymer was synthesized by polymerization between Fmoc-Asp and EGDE, with the modified procedure used in our previous study [21]. Briefly, Fmoc-Asp (5 mmol), EGDE (5 mmol), and TBAB (5 mg) were mixed with 1,4-dioxane (2.5 mL) and reacted at 100 °C for 48 h. After the reaction, synthesized FPED was precipitated by adding diethyl ether and dried under vacuum conditions. After that, the FPED was dissolved in 5 mL of DMF and activated by reaction with EDC (1 mmol), NHS (1 mmol), and DMAP (5 mg) for 1 h. Then, mPEG 750 (1 mmol) solution was prepared in DMF (1 mL) was prepared and added to the mixture. mPEGylation was carried out for 48 h. Thereafter, piperidine (4 mL) was added and stirred to remove Fmoc moiety. The deprotected mPEG-PED was precipitated by adding diethyl ether. The Fmoc-Arg (5 mmol) was reacted with EDC (5 mmol) and NHS (5 mmol) in DMF (10 mL) for 1 h. Activated Fmoc-Arg was added to dissolved mPEG-PED in DMF (5 mL) and reacted for 48 h. Then, piperidine (4 mL) was added and stirred to remove Fmoc moiety, precipitated by adding diethyl ether, and dried under vacuum. The final product mPEG-PEAD was dissolved in 0.1 M HCl (50 mL) and dialyzed using a dialysis tube (MWCO = 2000) against DW for 24 h. Dialyzed mPEG-PEAD was lyophilized and stored at −80 °C. These synthesized polymers were characterized via ^1^H-NMR.

### 4.4. Fabrication of TRAIL-Loaded mPEG-Coacervate

mPEG-PEAD and heparin were each dissolved in PBS at a concentration of 1 mg/mL and filtered through 0.2 μm syringe filters. Then, 1 μL of TRAIL solution (100 ng/μL) was mixed with 10 μL of prepared heparin solution. Subsequently, 80 μL of mPEG-PEAD solution was added to the heparin:TRAIL mixture (i.e., 1:100:800 mass ratios of TRAIL:heparin:mPEG-PEAD). An increase in turbidity was immediately observed, indicating the formation of mPEG-Coa. To observe morphological characteristics of mPEG-Coa, FITC-labeled BSA (FITC-BSA) instead of TRAIL was loaded in the mPEG-Coa, then TRAIL. Then, prepared FITC-BSA-loaded mPEG-Coa was observed using fluorescent microscopy (Nikon Ti-E, Tokyo, Japan).

### 4.5. Rheological Measurements

Rheological properties of mPEG-Coa were measured via Discovery HR-1 (TA instrument Inc., New Castle, DE, USA). mPEG-Coa was prepared as previously described and 200 μL of mPEG-Coa solution was loaded on the rheometer plate with a plate geometry of 20 mm radius. Frequency sweeps were conducted to 100 rad/s at a 0.01% of strain. Storage modulus and loss modulus measurements were performed in triplicate.

### 4.6. Release Kinetics of Cargo TRAIL form mPEG-Coacervate

mPEG-Coa containing 100 ng of TRAIL was fabricated as previously described. The prepared mPEG-Coa was then centrifuged at 12,000× *g* for 10 min to collect the supernatant. This supernatant was used to calculate TRAIL-loading efficiency. Subsequently, 500 μL of fresh PBS (135 mM of NaCl) or PBS additionally supplemented with 50 mM NaCl (185 mM of NaCl) was added to the mPEG-Coa pellet and resuspended. Resuspended samples were incubated at 37 °C for 14 days. At day 1, 3, 5, 7, and 14, supernatant was collected after centrifuge and resuspended with each fresh solution. Amounts of unloaded TRAIL (i.e., loading efficiency) and TRAIL released into the supernatant were measured using ELISA according to the manufacturer’s procedures.

### 4.7. Cargo TRAIL Protection Ability against Protease

After 100 ng of bolus TRAIL or 100 ng of entrapped cargo TRAIL in mPEG-Coa was incubated with 100 μL of 500 ng/mL trypsin at 37 °C for 10 h. 100 μL of 1× protease inhibitor solution was added to each trypsin-treated samples solutions and incubated for 5 min to halt digestion. After that, in order to remove TRAIL from mPEG-Coa, 50 μL of heparinase cocktail solution (1 μL of 4000 units/mL) was added and incubated at 37 °C for 1 h. The leftover TRAIL was quantified via ELISA.

### 4.8. Inhibition of Colon Cancer Recurrence Study

HCT-116 cells were seeded at a density of 2000 cells/well on a 96-well cell culture plate and incubated for 24 h. The old media were discarded and changed to fresh growth media containing 500 ng of bolus TRAIL or mPEG-Coa incorporated with 500 ng of cargo TRAIL. After TRAIL treatment, HCT-116 cells were incubated for 7 days. At day 3, 5, and 7, WST-1 assay was performed as previously described.

### 4.9. Statistical Analysis

Statistical analyses were conducted for quantitative data using GraphPad Prism 7.0 (GraphPad Software Inc., San Diego, CA, USA.). Quantitative experiments were performed in triplicate. Results were analyzed using one-way analysis of variance (ANOVA) and Tukey’s multiple-comparison test.

## Data Availability

Not applicable.
